# Towards accurate ^177^Lu SPECT activity quantification and standardization using lesion-to-background voxel ratio

**DOI:** 10.1186/s40658-023-00526-x

**Published:** 2023-01-23

**Authors:** Stanislav Raskin, Dan Gamliel, David Abookasis, Simona Ben-Haim, Alexandre Chicheportiche

**Affiliations:** 1 Department of Physics, Ariel University, Ariel, Israel; 2 Department of Electrical and Electronics Engineering, Ariel University, 407000 Ariel, Israel; 3 Department of Nuclear Medicine and Biophysics, Hadassah Medical Organization and Faculty of Medicine, Hebrew University of Jerusalem, 91120 Jerusalem, Israel; 4 University College London, London, UK

**Keywords:** ^177^Lu activity quantification, Gamma camera calibration, SPECT, Calibration factors, Sphere-to-background counts/voxel ratio

## Abstract

**Background:**

Conventional calibration of the gamma camera consists of the calculation of calibration factors (CFs) (ratio of counts/cc and true concentration activity) as the function of the volume of interest (VOI). However, such method shows inconsistent results when the background activity varies. The aim of the present study was to propose a new calibration method by considering the sphere-to-background counts/voxel ratio (SBVR) in addition to the VOI for CFs calculation. A PET cylindrical flood phantom, a NEMA IQ body phantom, a Data spectrum Torso Phantom (ECT/TOR/P) and a LK-S Kyoto Liver/Kidney phantom were used. The NEMA IQ phantom was used to calibrate the camera and to produce CFs for the different spheres volumes and for varying sphere-to-background activity ratios. The spheres were filled with a uniform activity concentration of ^177^Lu, while the background was first filled with cold water and activity was added between each SPECT scan. SPECT imaging was performed for 30-s, 20-s, and 10-s exposure per view. The calculated CFs were expressed as function of the sphere volume and SBVR. The obtained CFs were validated for an additional NEMA IQ acquisition with different activities in spheres and background and for the Torso and Liver/Kidney phantoms with inserted NEMA IQ spheres. The quantification accuracy was compared with the conventional method not taking SBVR into consideration.

**Results:**

The relative errors in quantification using the NEMA IQ phantom with the new calibration method were 0.16%, 5.77%, 9.34% for the large, medium and small sphere, respectively, for a time per view of 30-s. The conventional calibration method gave errors of 3.65%, 6.65%, 30.28% for 30-s. The LK-S Kyoto Liver/Kidney Phantom resulted in quantification errors of 3.40%, 2.14%, 11.18% for the large, medium and small spheres, respectively, for 30-s; compared to 11.31%, 17.54%, 14.43% for 30-s, respectively, for the conventional method. Similar results were obtained for shorter acquisitions times with 20-s and 10-s time per view.

**Conclusion:**

These results suggest that SBVR allows to improve quantification accuracy. The shorter time-per-view acquisitions had similar relative differences compared to the full-time acquisition which allows shorter imaging times with ^177^Lu and improved patient comfort. The SBVR method is simple to set up and can be proposed for standardization.

**Supplementary Information:**

The online version contains supplementary material available at 10.1186/s40658-023-00526-x.

## Background

The radioisotope Lutetium 177 (^177^Lu) is increasingly used in nuclear medicine clinics thanks to its useful applications in molecular radiotherapy and especially in peptide receptor radionuclide therapy (PRRT) [1–3]. ^177^Lu emits short-range therapeutic beta particles and gamma photons with energies of 113 keV and 208 keV allowing for quantitative imaging and personalized dosimetry calculations [4–9]. With PRRT, the aim is to obtain an optimal therapeutic effect to tumors without exceeding safety absorbed dose thresholds to the healthy organs, mainly kidneys and bone marrow. In our center, dosimetry is performed after each PRRT cycle and therapy is stopped once the cumulative dose absorbed by kidneys and bone marrow is expected to exceed 25 Gy and 2 Gy, respectively [5, 6]. A recent phase II trial showed the superiority of patient individualized dosimetry-based PRRT treatments in terms of the efficacy and safety [10], highlighting the importance of accurate dosimetry calculations for PRRT. However, the accuracy of the absorbed dose estimations greatly depends on the quantification process. Imaging quantification is a multi-step process which translates a SPECT/CT detected signal (counts) into radiotracer activity. Activity quantification from SPECT images is not a trivial task. Inaccuracies in image reconstruction and thus in radionuclide uptake estimates are introduced through various factors such as photon attenuation and scatter, gamma camera dead time, collimator blurring and reconstruction algorithms which may cause image artifacts and image degradation and, the partial volume effect (PVE), inherited by equipment and software limitations.

The conversion of photon counts to activity is accomplished by calibration of the gamma camera. The gamma camera calibration factor (CF) depends on the type of collimator, camera spatial resolution, camera sensitivity, peak and scatter acquisition windows and reconstruction algorithms. Reconstruction techniques such as ordered subset expectation maximization (OSEM) allow for attenuation correction using CT, for scatter correction using double (DEW) or triple energy window (TEW) and resolution recovery enables collimator-detector response compensation.

Conventional gamma camera calibration [11–15] is performed using a simple cylindrical phantom with spherical inserts filled with uniform activity concentration while the background is filled with cold or hot water, in order to simulate lesions and approximate the clinical conditions of a real patient. Using image processing software, phantom spheres are delineated as volumes of interest (VOIs), and a CF is calculated as the ratio between counts/cc in sphere VOIs and the true activity concentration for different sphere volumes. However, this method causes inconsistencies between repeated measurements of the same reference phantom with different background activity concentrations. The impact of the background activity concentration on the CFs is well known; however, to the best of our knowledge, little effort went into correcting for its actual effects [13, 16–18]. De Nijs et al. [13] reported that small changes in the background to sphere activity ratio may influence the obtained CFs, especially for small volumes of interest (VOI). 

The aim of the present study was to propose and validate a new calibration method using the lesion-to-background counts/voxel ratio and the lesion volume as varying parameters for determination of the camera CFs, improving quantification accuracy.

## Methods

### Phantom preparation

**Fig. 1:**
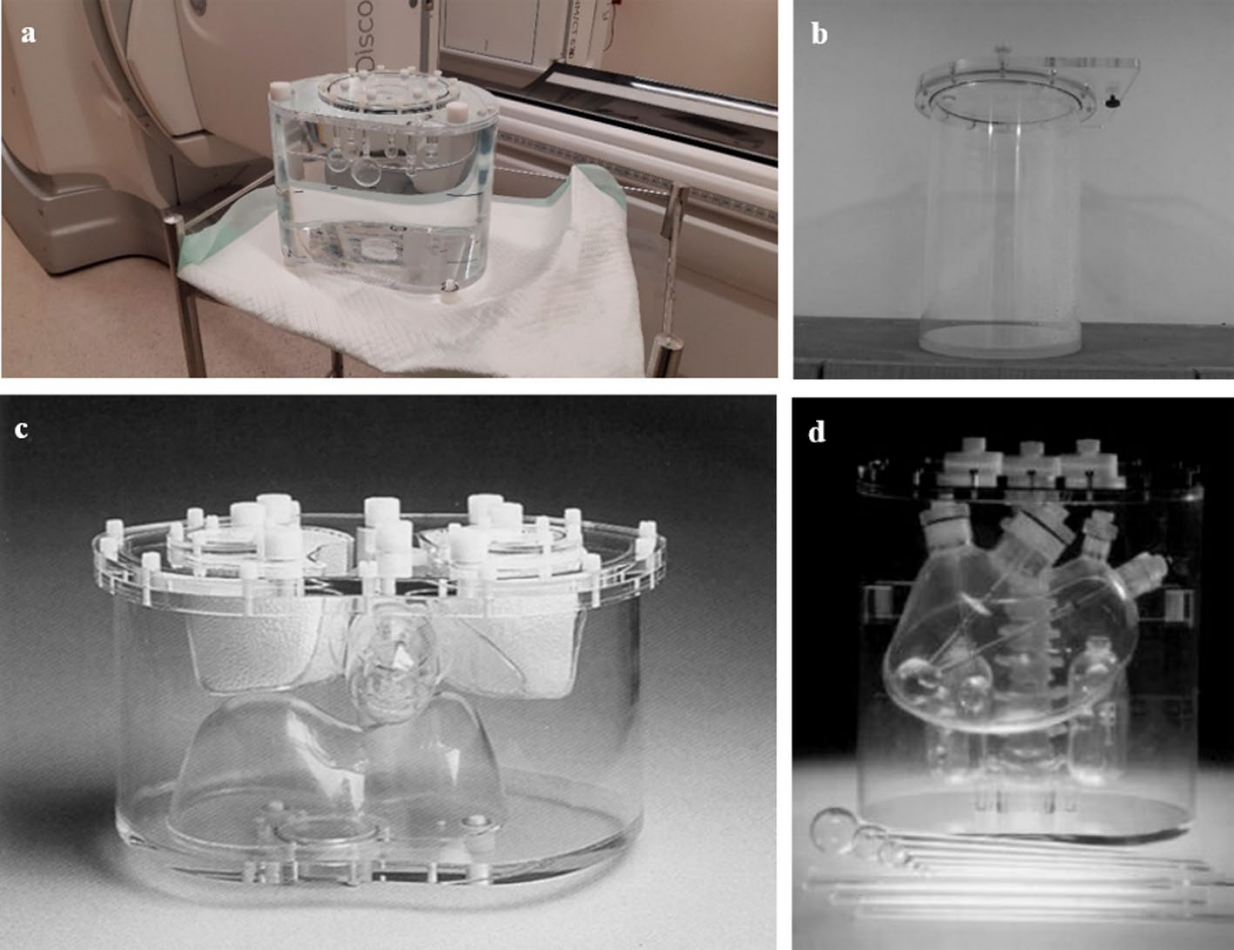
**a** NEMA IQ phantom and **b** PET cylindrical flood phantom used for calibration. Anthropomorphic **c** Torso (ECT/TOR/P, (Data Spectrum, Hillsborough, NC)) and **d** LK-S Kyoto Liver/Kidney phantoms used for ^177^Lu activity quantification validation in spheres and large organs

Several phantoms were used in this study. A PET cylindrical flood phantom and a NEMA image quality (IQ) body phantom (Model PET/IEC-BODY/P) were used for calibration and validation purposes, while the anthropomorphic Torso (ECT/TOR/P, (Data Spectrum, Hillsborough, NC)) and LK-S Kyoto Liver/Kidney phantoms were used for validation and quantification accuracy estimation (Fig. 1).

The PET cylindrical flood phantom was filled with an activity of 1.4 GBq of ^177^Lu-DOTATATE leading to an activity concentration of 0.25 MBq/mL. This phantom was used to calculate a CF from a uniform activity distribution [14] following the conventional method. The NEMA IQ phantom was used for both calibration and validation. This phantom contains six fillable spheres with volumes of 26.52 cc, 11.49 cc, 5.71 cc, 2.84 cc, 1.23 cc and 0.44 cc and a background compartment of 9.7 Liter. This phantom was first used to determine CFs for different sphere volumes according to the conventional method (NEMA Cal. 1 acquisition). The spheres contained a uniform ^177^Lu-DOTATATE activity concentration of 2.62 ± 0.04 MBq/cc, while the background has a concentration activity of 0.16 MBq/cc. Secondly, the NEMA IQ phantom was prepared with varying surrounding background activity to assess the effect of the background activity (NEMA Cal. 2 acquisition). The six spheres were filled with a uniform ^177^Lu-DOTATATE activity concentration of 1.89 ± 0.04 MBq/cc, and the phantom was scanned with five different background activity concentrations (0, 0.11, 0.14, 0.19 and 0.31 MBq/cc). CFs were calculated for all the different configurations. One additional NEMA IQ phantom was prepared for validation purpose with spheres activity concentration of 2.14 ± 0.01 MBq/cc and a background activity concentration of 0.14 MBq/cc (NEMA Val. acquisition).

The anthropomorphic Torso Phantom (Torso Val. phantom) simulates the upper torso of average subjects and was used for quantification accuracy validation. The phantom includes left and right lungs filled with polystyrene and water to simulate lung tissue density, a 1.2-L liver fillable compartment, a background region and spine. The 26.52 cc and 5.71 cc NEMA IQ spheres filled with ^177^Lu-DOTATATE activity concentration of 2.66 MBq/cc were inserted in the 0.26 MBq/cc liver and 0.03 MBq/cc background regions, respectively. The anthropomorphic LK-S Kyoto Liver/Kidney phantom (Liver/Kidney Val. Phantom) was used to assess the quantification accuracy for large organs, i.e., liver and kidneys, and for lesions. The phantom was prepared by inserting the 26.52 cc, 11.49 cc and 5.71 cc NEMA IQ spheres with a respective ^177^Lu-DOTATATE activity concentration of 6.14 MBq/cc, 4.77 MBq/cc and 3.56 MBq/cc in different regions of the anthropomorphic phantom. The 26.52 cc and 5.71 cc spheres were inserted in the 0.27 MBq/cc liver compartment, and the 11.49 cc sphere was inserted in the 0.08 MBq/cc background compartment. The left and right kidneys contained a ^177^Lu activity concentration of 0.63 MBq/cc and 0.89 MBq/cc, respectively. Table 1 summarizes the ^177^Lu activity concentrations contained in the different phantoms and spheres.

**Table 1:** Phantom acquisitions and configurations

Phantom	26.52 [cc]	11.49 [cc]	5.71 [cc]	Liver	Kidneys [L/R]	Background cavity
*Activity concentration [MBq/cc]*						
PET cylindrical	–	–	–	–	–	0.25
NEMA Cal. 1	2.67	2.63	2.58	–	–	0.16
NEMA Cal. 2. (5 acquisitions)	1.94	1.87	1.82	–	–	0, 0.11, 0.14, 0.19, 0.31
NEMA Val.	2.14	2.14	2.14	–	–	0.14
Torso Val.	2.66	–	2.66	0.26		0.03
Liver/Kidney Val.	6.14	4.77	3.56	0.27	0.63/0.89	0.08

### Image acquisition and reconstruction

All phantom acquisitions were performed using a Discovery NM/CT 670 (International General Electric, General Electric Medical Systems, Haifa, Israel) gamma camera. This system combines a dual-head coincidence SPECT camera with an axial field of view (FOV) of 40 × 54 cm, a 9.5-mm-thick NaI(Tl) crystal, and 59 photomultiplier tubes (PMT). All SPECT images were acquired with a 20% energy window around the main photopeak of ^177^Lu (208 keV; 11% probability) with medium-energy general purpose (MEGP) collimators. The different acquisitions were performed by applying 60 views over 360° (30 angular steps per head, 6° angle step) with a 30-s exposure per frame (15 min acquisition) in a 128 × 128 matrix size (4.4 mm pixels). In addition, for the NEMA Val., Torso Val. and Liver/Kidney Val. phantoms, 20-s and 10-s per frame acquisitions were obtained.

Image reconstruction was performed using the General Electric (GE) Dosimetry toolkit (DTK) software [19] available for the Xeleris 3.0 Workstation (International General Electric, General Electric Medical Systems, Haifa, Israel). The ordered subsets expectation maximization (OSEM) algorithm with a subset and iteration product of 100 (10 iteration and 10 subsets) was used in order to obtain counts convergence in small VOIs [12]. Additionally, attenuation correction (from CT attenuation maps) and resolution recovery (for reducing image blurring) included in the Xeleris 3.0 workstation were used. For scatter correction, the dual energy window (DEW) method was used. This method consists of measuring the scatter in an energy window juxtaposed just below the main photopeak window (208 keV) that was placed ± 10% around 166.4 keV [20]. Then, a pixel-by-pixel correction subtracting the scatter counts from the main photopeak counts was performed. This correction uses a weighting factor, which depends on the width of the main peak and scatter energy windows [6].

### Image analysis

**Fig. 2:**
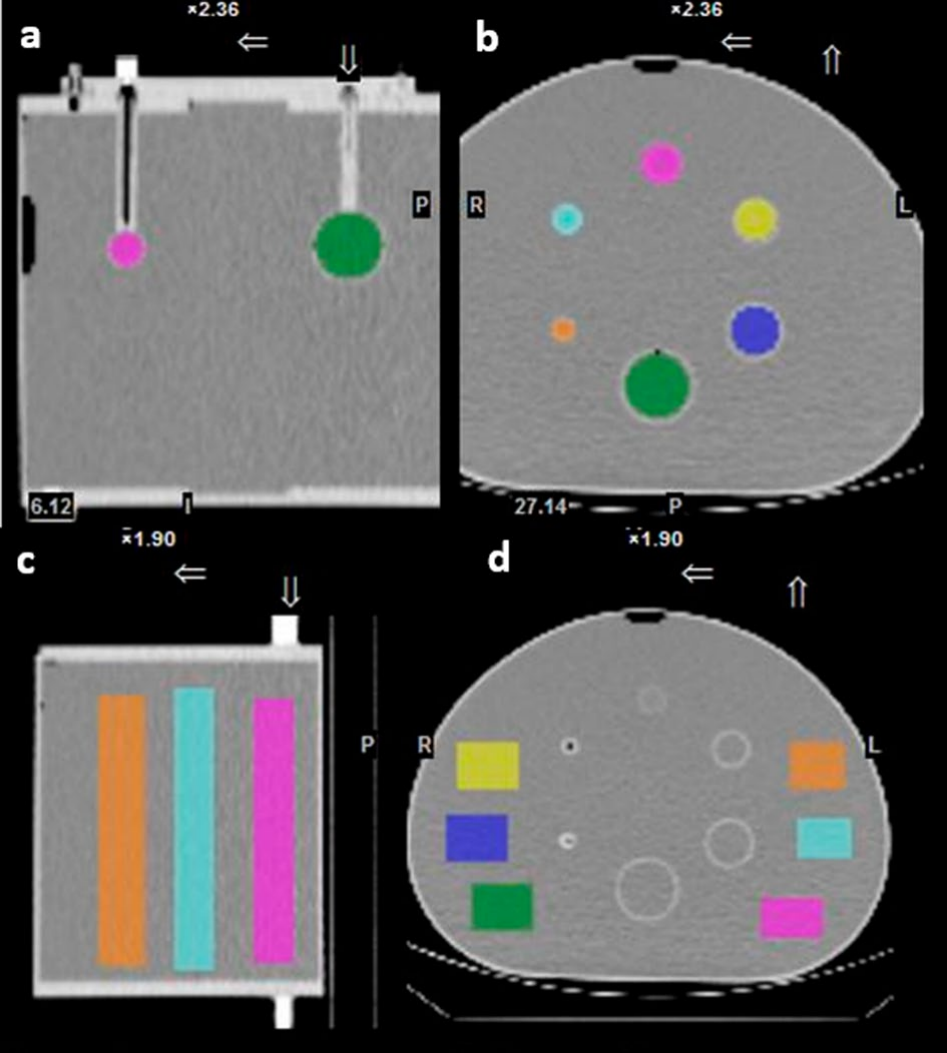
**a**, **b** Spheres and **c**, **d** background volumes of interest (VOIs) drawn on the NEMA IQ Cal. phantom using the General Electric Dosimetry toolkit (GE DTK) software

Processing in GE DTK includes either semi-automatic (threshold approach) or manual three-dimensional delineation of the VOIs on SPECT or CT images. For the PET cylindrical phantom, the semi-automatic delineation tool was used on the SPECT image to delineate the whole phantom activity. For determination of background CF in the NEMA Cal. 2 acquisition, six rectangular VOIs were drawn manually across the whole length of the phantoms background. The sphere VOIs in the different phantoms (NEMA IQ, Torso Val. and Liver/Kidney Val.) were drawn manually on CT images using the DTK sphere tool. For liver and kidneys in the anthropomorphic phantoms, VOIs were drawn manually on CT images and were placed over the whole organs. It is noteworthy that the GE DTK software does not allow copying the VOIs delineated on a SPECT/CT acquisition to another study. Therefore, all sphere VOIs were drawn again on attenuation SPECT images for each acquisition (by the same user). Figure 2 shows an example of the drawn sphere and background VOIs using the GE DTK software. Finally, as output, the GE DTK software gives the volume of the VOIs, the total number of counts and the number of counts per voxel in the drawn VOIs.

### Calibration factors calculation

The CFs [cps/MBq] for both methods were calculated using the following formula:

1 $${\text{CF}} = \frac{{{\text{Cts}}_{{{\text{voi}}}} }}{{T_{{{\text{acq}}}} *V_{{{\text{voi}}}} *C_{{{\text{true}}}} }}$$

where ${\text{Cts}}_{{{\text{voi}}}}$ is the total counts in the delineated VOI, $V_{{{\text{voi}}}}$ is volume [cc] of the VOI, $C_{{{\text{true}}}}$ is the calibrated activity concentration [MBq/cc] in the VOI region, and $T_{{{\text{acq}}}}$ is total image acquisition time in sec (product of the time per view and number of views).

#### Calibration factors—conventional method

*Background/Organ calibration factors* The background CF for the conventional method is used to quantify large organs (kidneys and liver) and was calculated from the PET cylinder acquisition by delineating the whole phantom volume and using Eq. 1.

*Sphere/lesions calibration factors* CFs for the three largest spheres of the NEMA Cal. 1 acquisition were calculated using Eq. 1.

#### Calibration factors—SBVR method

*Background calibration factors* The background CF for the SBVR method was calculated using an alternative method with the aim to simplify the entire calibration process by using a single phantom (i.e., NEMA IQ). The background CF was obtained from each of the four NEMA Cal. 2 acquisitions with hot background by averaging the CFs obtained in the six rectangular VOIs as described above. In total, four background CFs were obtained from the NEMA Cal. 2 across the five acquisitions. (Background CF was not calculated for the NEMA Cal. 2 acquisition with cold background.) The four background CFs were averaged and the resulting background CF obtained with the SBVR method was compared to the conventional background CF.

*Sphere/lesions calibration factors* The CFs for the three largest spheres of the NEMA Cal. 2 acquisition (26.52, 11.49 and 5.71 cc) were determined from the five acquisitions from cold to hot background (range 0–0.31 MBq/cc) using Eq. 1, as described before (Table 1). In total, 15 CFs were calculated for the five different NEMA Cal. 2 acquisitions.

For a given sphere, the CFs varied in the different acquisitions as the function of the background concentration activity. Therefore, CFs were expressed as function of the sphere-to-background activity concentration ratio (SBAR of ∞:1, 17:1, 14:1 10:1 and 6:1, respectively, to the 0, 0.11, 0.14, 0.19 and 0.31 MBq/cc background), as well as the sphere volume. In addition, the CFs were expressed as function of sphere-to-background voxel ratio (SBVR) as well as the sphere VOI volume.

*Sphere-to-Background Voxel Ratio (SBVR)* Sphere-to-background voxel ratio (SBVR) was defined as the ratio between the total counts per voxel in a VOI and the total counts per voxel in a surrounding background VOI, as:

2 $${\text{SBVR}} = \frac{{{\text{Ctspv}}_{{{\text{VOI}}}} }}{{{\text{Ctspv}}_{{{\text{Background}}}} }}$$

Here, ${\text{Ctspv}}_{{{\text{VOI}}}}$ is the total counts per voxel in a VOI and ${\text{Ctspv}}_{{{\text{Background}}}}$ is the total counts per voxel in the background VOI. The SBVR was calculated for each of the three largest spheres and for every NEMA Cal. 2 acquisition. The total counts per voxel in the background were calculated by averaging the total counts per voxel in each of the six rectangle VOIs in each acquisition. In total, 12 SBVRs were calculated for the three largest spheres across the four NEMA Cal. 2 acquisitions with hot background. In order to assess the effects of the background on CFs for all background activity concentrations, CFs were plotted against SBVR as a linear function for each of the three spheres separately. For cold background, the spheres SBVR values could not be calculated directly using Eq. (2) due to the absence of activity in the background (SBVR values of infinity). Instead, the SBVR value leading to a maximum CF for each sphere was obtained by determining the point of intersection between the CF versus SBVR linear curve and the cold background CF value for each of the three spheres.

To obtain the CF versus SBVR calibration curves for a larger series of volumes, the CFs were expressed as function of the VOI volume by exponentially fitting the CFs obtained for the three spheres (from the linear functions) and for a fourth large VOI. The latter was calculated for different SBVR values using a transition factor (TF) calculated from cold background. TF was calculated by dividing a background CF value - CF_BG_, where the PVE is negligible and there are no background effects, by the CF of the largest sphere in the cold background acquisition - $CF_{{26.52\left( {{\text{cold}}} \right)}}$ as follows:

3 $${\text{TF}} = \frac{{{\text{CF}}_{{{\text{BG}}}} }}{{{\text{CF}}_{{26.52\left( {{\text{cold}}} \right)}} }}$$

Therefore assuming that TF stays constant for all background activity concentrations, a large volume CF for a given SBVR is obtained by multiplying the corresponding CF_26.52_ by TF. Combining both CF versus SBVR and CF versus VOI volume curves resulted in the mapping of the CFs as function of SBVR and the VOI (lesion) volume.

### Validation of quantitative imaging

**Fig. 3:**
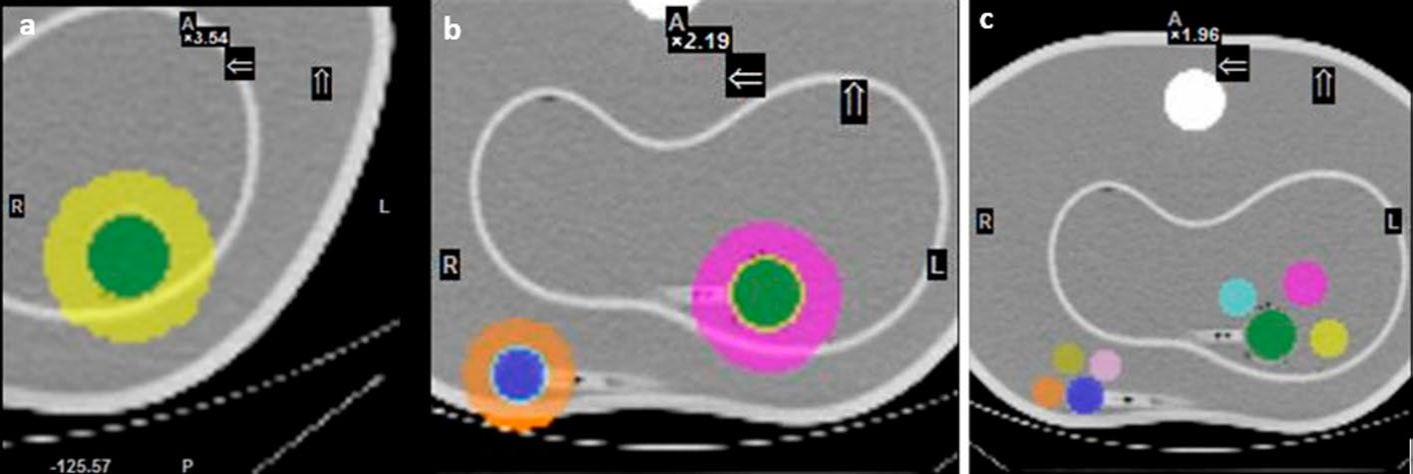
Delineation methods for anthropomorphic phantoms. In methods **a** 1 and **b** 2, the inner VOI (green or blue) represents the sphere VOI, while the outer thick ring delineates the surrounding background VOI. In method 2 (**b**), the thin middle ring is used to separate both VOIs to prevent spill in/out from sphere to background. With method 3 (**c**), random background spherical VOIs are placed around the sphere VOI

Validation of the conventional and SBVR calibration methods was performed using the NEMA Val., Torso Val. and Liver/Kidney Val. acquisitions. The conventional quantification method [12, 21] uses the sphere CFs calculated from NEMA Cal. 1 acquisition and the background CF calculated from PET cylindrical phantom for quantification of the large organs, without taking background effects into consideration. For the SBVR method, quantification was done using the CF map. The SBVR and VOI volume values were obtained from delineation of the spheres, liver, and kidneys and their surrounding background in the anthropomorphic and NEMA IQ (NEMA Val. acquisition) phantom acquisitions. Both values (VOI volume and SBVR) were then plugged into the CF map to obtain a corresponding CF which is used for quantification. Moreover, SBVR values were calculated using different background delineation methods due to the non-uniformity of the background around the spheres and around the large organs (liver and kidneys). For example, Fig. 3 shows different configurations where spheres are close to two different background regions. Indeed, Fig. 3b shows that the large sphere (green) is surrounded by both the liver activity and the phantom background activity. For the small sphere (blue), the effects of the surrounding activities on the quantification may be even more important since the latter is surrounded by phantom background activity on one hand and by air (null activity) on the other hand. To address this problem, three delineation methods (Fig. 3a–c) were tested. In the first method (Fig. 3a), a thick background ring was placed around the sphere VOI. The second method (Fig. 3b) includes an additional thin separation ring placed between the sphere VOI and the background ring to eliminate spillover from the sphere VOI to the background. In the third method (Fig. 3c), random background spherical VOIs were placed around the sphere VOI and the counts per voxel were calculated as the average across the background spherical VOIs. Validation was also performed for the different time-per-view settings for the NEMA Val., Torso Val. and Liver/Kidney Val acquisitions.

### Statistical analysis

The CF versus SBVR curves were fitted using a linear fit. The CF versus VOI volumes were fit using an exponential fit. The best fit has been found to be with the following structure:

$$f\left( x \right) = Ae^{bx} + Ce^{dx}$$

CFs linear and exponential fitting was performed using MATLAB (MATLAB Release 2022a, The MathWorks, Inc., Natick, MA, USA).

Comparison between the conventional, SBAR and SBVR methods was assessed by comparing the quantification accuracy of the different methods as the relative difference between the true injected activity concentration in the spheres/organs and the estimated activity concentrations using the different calibration methods.

## Results

### Calibration factors—conventional method

*Conventional Background Calibration Factor* The background CF obtained from the PET cylindrical flood phantom acquisition was 5.00.

*Conventional Sphere Calibration Factors* Using the NEMA Cal. 1 acquisition, CFs of 3.92, 3.18 and 2.64 ﻿cps/MBq were obtained for the 26.52, 11.49 and 5.71 cc spheres, respectively.

### Calibration factors—SBVR method

*Background Calibration Factor* The background CF for the SBVR method was obtained from the four NEMA Cal. 2 acquisitions with background activity concentrations of 0.11, 0.14, 0.19 and 0.31 MBq/cc. Corresponding background CFs of 5.08, 5.00, 4.75 and 4.66 ﻿cps/MBq were obtained, respectively, with a mean average background CF value of 4.87 leading to a mean relative difference of 2.6% from the PET cylindrical flood phantom CF of 5.00. This promoted us to use the background CF obtained from the SBVR method using the NEMA Cal. 2 acquisition (4.87 ﻿cps/MBq) instead of the one obtained using the PET cylinder acquisition (5.00 ﻿cps/MBq). As such, all CFs for the SBVR method could be calculated using the NEMA IQ phantom. Moreover, the calculation of the TF has been done using the SBVR background CF instead of the conventional background CF (which fulfills negligible PVE and no background effects conditions).

**Fig. 4:**
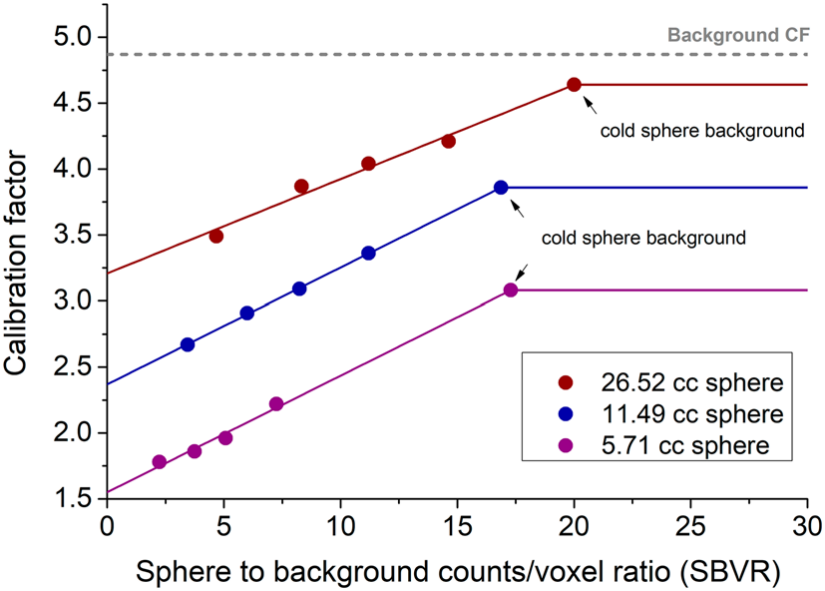
CF ﻿values in cps/MBq versus SBVR calibration curves for the 26.52 (red curve), 11.49 (blue curve) and 5.71 cc (purple curve) spheres. The CFs data obtained from several NEMA Cal. 2 acquisitions (dots) were interpolated using a simple a linear regression fit. The “cold background” points were obtained from the point of intersection between the linear curve and the cold background CF value for each of the three spheres. The cold background CF value represents the maximum value of the CF for every sphere. The horizontal gray dashed line with a value of 4.87 represents the background CF for large volumes

*Sphere Calibration Factors using SBVR* For each sphere and each NEMA Cal. 2 phantom acquisition with hot background, CFs and SBVR values were calculated. CFs values of 4.21, 3.39 and 2.22 ﻿cps/MBq and SBVRs of 14.63, 11.20 and 7.25 were obtained for the 0.11 MBq/cc background for the 26.52, 11.49, and 5.71 cc spheres, respectively. For the 0.14 MBq/cc background, CFs of 4.04, 3.09 and 1.96 ﻿cps/MBq were obtained for SBVR values of 11.19, 8.24 and 5.08, respectively. For the 0.19 MBq/cc and 0.31 MBq/cc background, CFs of 3.87, 2.91 and 1.82 and, 3.49, 2.67 and 1.78 ﻿cps/MBq and SBVR values of 8.33, 5.99 and 3.75 and, 4.68, 3.46 and 2.43, respectively, were obtained. For the cold background acquisition, CFs of 4.64, 3.86 and 3.08 ﻿cps/MBq were obtained with SBVR values of 20.01, 16.87 and 17.29, respectively. Figure 4 and Additional file 1: Table S1 summarize the CF and SBVR values for the 26.52, 11.49, and 5.71 cc spheres. As described before, to express the CFs for continuous SBVR values, the CFs data were interpolated using a simple linear regression fit. Figure 4 shows an excellent correlation between the CF and SBVR values with Pearson’s *r* of 0.99 for all three spheres. For SBVR values higher than those obtained for the cold background acquisition, we supposed that the CFs reached their maximum and do not vary anymore.

**Fig. 5:**
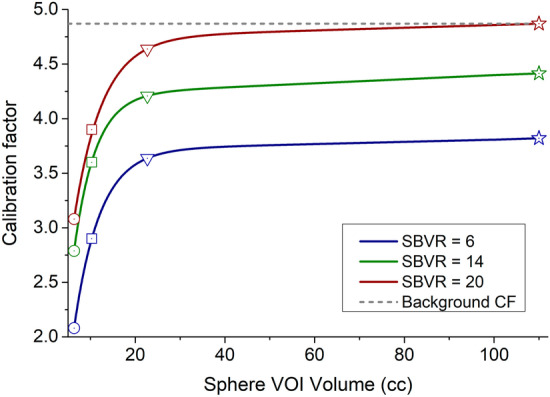
CF values in cps/MBq versus sphere/lesion volume curves for SBVR values of 6 (blue curve), 14 (green curve) and 20 (red curve). The gray dashed line represents the maximum CF (4.87). The circle, square, triangle symbols show the know CF values for the 5.71, 11.49 and 26.52 cc spheres, respectively, from Fig. 4. The star symbol represents the CF value calculated from the transition factor TF (Eq. 3) for a sphere/lesion volume of 110 cc

*Calibration Factors Map as function of SBVR and sphere volume* The calibration curves presented above account for only three sphere/lesion volumes (26.52 cc, 11.49 cc and 5.71 cc). SBVR versus CF curves have been extended to all lesion volumes. For a given SBVR, the CF values obtained for the three spheres and for a large VOI volume were plotted as a function of the spheres volume and then interpolated using an exponential fit. Figure 5 shows the exponential fit of the CFs values as function of the VOI volume for SBVR values of 6, 14 and 20.

**Fig. 6:**
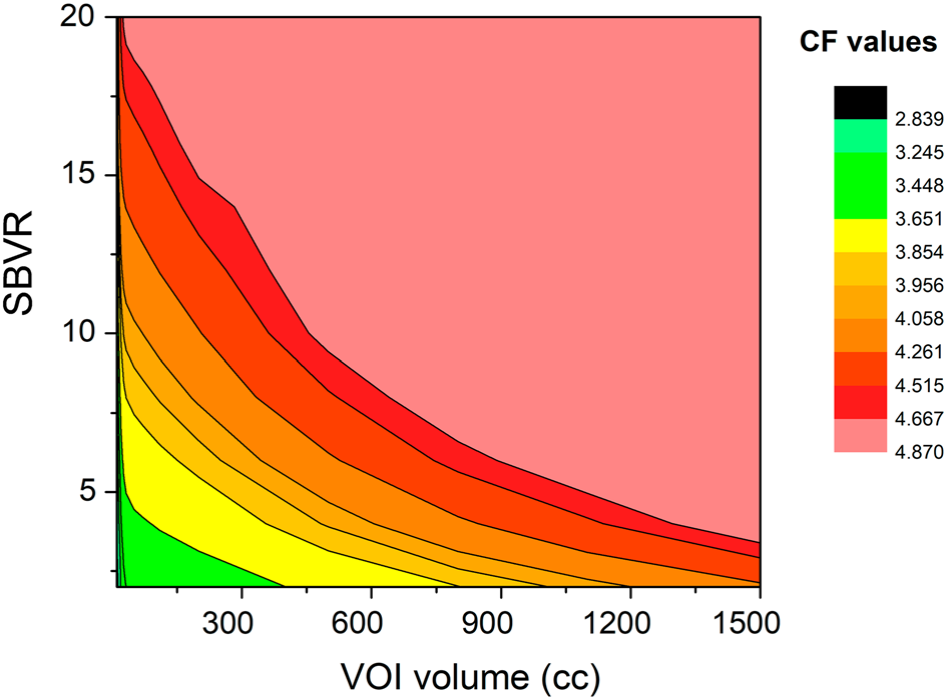
Calibration factor map [﻿cps/MBq] as function of sphere-to-background counts/voxel ratio (SBVR) and VOI volume [cc]

CFs versus volume fits were performed for SBVR values ranging from 2 to 20. The resulting data were organized into a Calibration Factor map, providing the CF values for a given SBVR between 2 and 20 and a given VOI volume between 6 and 1500 cc (cf. Figure 6 and Additional file 1: Table S2).

### Validation of quantitative imaging

*Validation of the quantification using the NEMA IQ body phantom* The CFs obtained with the conventional calibration method, i.e., NEMA Cal. 1, were applied to the NEMA Val. acquisition with the background CF used to quantify the liver and kidneys and sphere CFs for the spheres. Mean relative deviations from the true concentration activity of − 3.65% for the 26.52 cc sphere, 6.65% for the 11.49 cc sphere and of 30.28% for the 5.71 cc sphere for the 30-s per frame acquisition were obtained. For the 20-s per frame, mean relative errors of − 1.78% for the 26.52 cc sphere, 9.69% for the 11.49 cc sphere and of 29.19% for the 5.71 cc sphere were obtained. For 10-s per frame mean relative errors of 1.39% for the 26.52 cc sphere, 4.48% for the 11.49 cc sphere and of 24.32% for the 5.71 cc sphere were obtained.

There were inconsistencies in the SBAR since a similar SBAR ratio led to different CFs. Indeed, as shown in Table 2, for a comparable sphere to SBAR, the difference in the CFs for the smallest sphere results in a significant error up to 30.3%.

**Table 2:** Comparison between CFs calculated from acquisitions NEMA Cal.1 and NEMA Val. for comparable sphere–background activity ratios (SBARs)

Sphere volume (VOI volume) [cc]	Phantom NEMA Cal. 1 acquisition		Phantom NEMA Val. acquisition		CF difference error (%) between NEMA Cal 1. and NEMA Val.
	CF [﻿cps/MBq]	SBAR	CF [﻿cps/MBq]	SBAR	
26.52 (22.7 cc)	3.92	15.91	4.06	15.28	3.44
11.49 (10.3 cc)	3.19	15.91	2.96	15.28	7.21
5.71 (6.36 cc)	2.64	15.91	1.84	15.28	30.30

*CF*, Calibration factor; *SBAR*, Sphere-to-background counts/activity ratio

Consequently, the SBVR method was tested on NEMA Val. phantom. A SBVR value was calculated for each of the three largest spheres. Using the CF map (Fig. 6 and Additional file 1: Table S2), CFs of 4.07, 3.15 and 2.03 ﻿cps/MBq were obtained for the 26.52, 11.49 and 5.71 cc spheres. These CF values led to mean relative deviations from true concentration activity of 0.16%, 5.77% and 9.34%, respectively, for the 30-s per frame exposure. For 20-s time per view, respective relative deviations for true activities of 1.72%, 8.25% and 8.84% were obtained. For 10-s respective relative deviations for true activities of 4.56%, 4.48% and 4.41% were obtained. The quantification errors for both the conventional method and the SBVR method for the different time per view settings are summarized in Table 3.

**Table 3:** Quantification errors between activity concentrations calculated with the conventional method and with the SBVR method for different time per view settings (minus sign – overestimation)

Sphere volume (VOI volume) [cc]	Phantom NEMA Val. acquisition validation					
	Quantification error (%)					
	Time per view: 30-s		Time per view: 20-s		Time per view: 10-s	
	SBVR Method (CF/ SBVR)	Conv. Method (CF)	SBVR Method (CF/ SBVR)	Conv. Method (CF)	SBVR Method (CF/ SBVR)	Conv. Method (CF)
26.52 (22.7 cc)	0.16% (4.07/12.16)	− 3.65% (3.92)	1.72% (4.07/12.07)	− 1.78% (3.92)	4.56% (4.05/11.82)	1.39% (3.92)
11.49 (10.3 cc)	5.77% (3.15/8.90)	6.65% (3.19)	8.25% (3.13/8.71)	9.69% (3.19)	4.48% (3.19/9.32)	4.48% (3.19)
5.71 (6.36 cc)	9.34% (2.03/5.53)	30.28% (2.64)	8.84% (2.05/5.67)	29.19% (2.64)	4.41% (2.09/6.11)	24.32% (2.64)

*VOI*, Volume of interest; *CF*, Calibration factor [﻿cps/MBq]; and *SBVR*, Sphere-to-background counts/voxel ratio

**Fig. 7:**
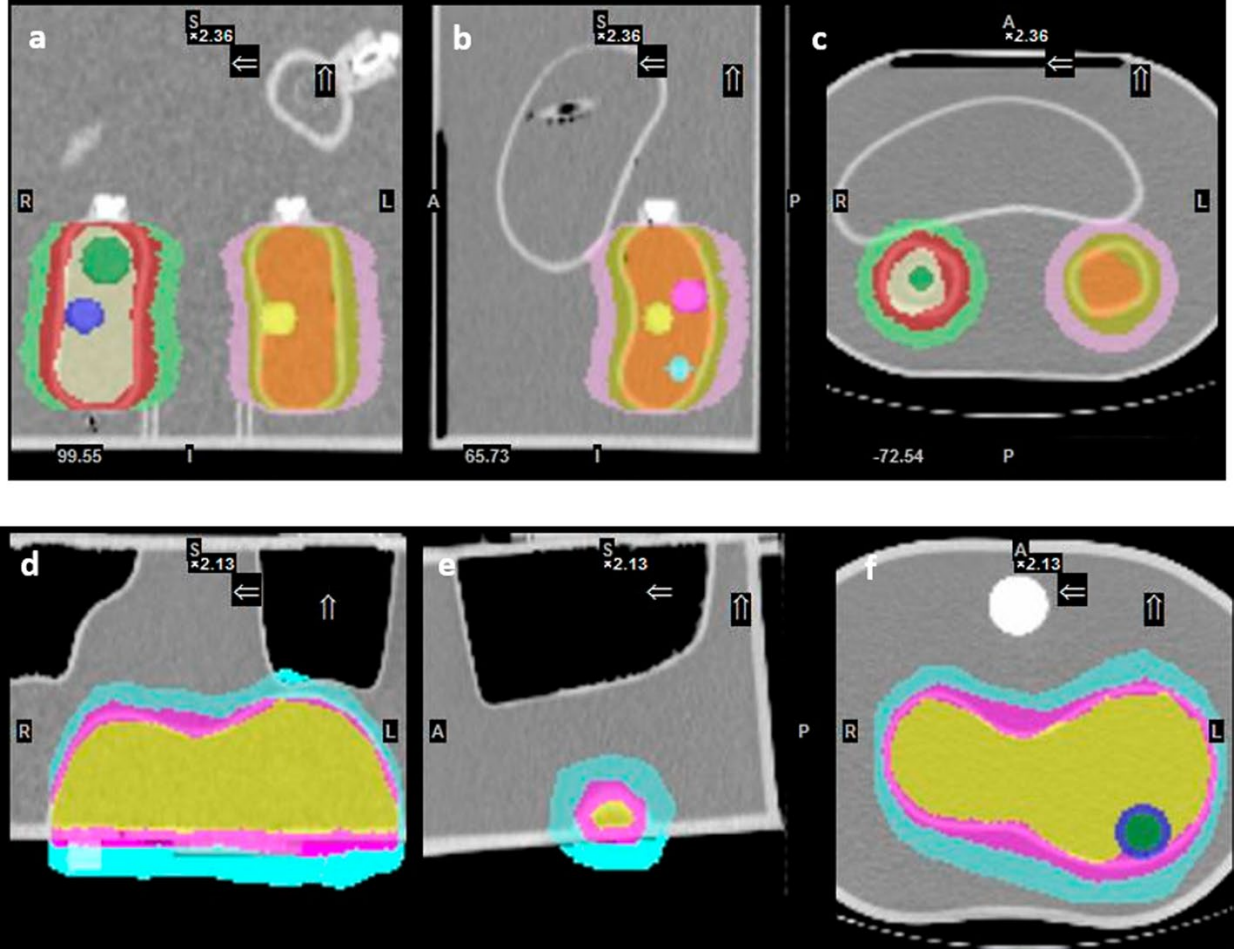
Delineation methods for the large organs in the anthropomorphic phantoms. The top images (**a**–**c**) show the delineation of the kidneys in the liver/kidney phantom, and the bottom images (**d**–**f**) show the delineation of the liver cavity in the torso phantom

*Validation of the quantification using anthropomorphic phantoms* The Torso Val. and Kidney/Liver Phantom acquisitions were used to validate the SBVR method. A comparison with the conventional method was also performed. For the SBVR method, delineation of the background VOI for both acquisitions was done using the different methods described above. The method consisting of randomly placing a spherical background VOI adjacent to the sphere VOIs proved to be inconsistent, with a large difference in quantification accuracy for the different spheres (12–18% error). The method using a separation ring between the sphere VOI and the background ring provided the best quantification accuracy (6–7% error vs 1–18% without separation ring) and was chosen for calculation of SBVR values and validation of the quantification with the anthropomorphic phantoms (Fig. 7).

Using the CF map, SBVR CFs of 4.24, 2.98 and 4.87 ﻿cps/MBq were obtained for the 26.52 and 5.71 cc spheres and for the liver in the Torso Val. phantom. Mean relative deviations from true concentration activity of − 7.30%, − 9.34% and − 1.50% were, respectively, obtained compared to − 16.06%, − 23.42% and 1.10% with the conventional method, respectively, for the 30-s per frame exposure. With the Kidney/Liver Val. phantom, mean relative deviations of 3.40% for the 26.52 cc, 2.14% for the 11.49 cc and − 11.88% for the 5.71 cc spheres, − 1.35% for liver and 3.00% for the right and − 5.06% left kidneys were obtained with the SBVR method compared to − 11.31%, − 17.54%, − 14.43%, 11.86%, 26.32% and 20.98% with the conventional method, respectively, for the 30-s per frame exposure. The quantification errors for the conventional and the SBVR methods for the different time per view settings (30-s, 20-s and 10-s time per view) are shown in Tables 4 and 5 for the Torso Val. and for the Kidney/Liver Phantom acquisitions, respectively.

**Table 4:** Quantification errors between activity concentrations calculated with the conventional method and the SBVR method for different time per view settings for spheres and liver in the Torso Phantom (minus sign – overestimation)

Organ/Sphere volume (VOI volume) [cc]	Torso Val. Phantom acquisition validation					
	Quantification error (%)					
	Time per view: 30-s		Time per view: 20-s		Time per view: 10-s	
	SBVR Method (CF/ SBVR)	Conv. Method (CF)	SBVR Method (CF/ SBVR)	Conv. Method (CF)	SBVR Method (CF/ SBVR)	Conv. Method (CF)
26.52 (22.7 cc)	− 7.30% (4.24/1.50)	− 16.06% (3.92)	− 8.14% (4.09/12.43)	− 12.83% (3.92)	− 10.07% (3.92/9.93)	− 10.63% (3.92)
5.71 (6.36 cc)	− 9.34% (2.98/63.90)	− 23.42% (2.64)	− 8.18% (2.98/105.61)	− 26.21% (2.64)	− 10.18% (2.98/70.61)	− 24.73% (2.64)
Liver (~ 1200 cc)	− 1.50% (4.87/15.0)	1.10% (5.0)	3.87% (4.87/12.0)	6.34% (5.0)	3.31% (4.87/11.0)	5.82% (5.0)

*VOI*, Volume of interest; *CF*, Calibration factor [﻿cps/MBq]; and *SBVR*, Sphere-to-background counts/voxel ratio

**Table 5:** Quantification errors between activity concentrations calculated with the conventional method and the SBVR method for different time per view settings for spheres, kidneys and liver in the L-KS Phantom (minus sign – overestimation)

Organ/Sphere volume (VOI volume) [cc]	Kidney/Liver phantom Val. acquisition validation					
	Quantification error (%)					
	Time per view: 30-s		Time per view: 20-s		Time per view: 10-s	
	SBVR Method (CF/ SBVR)	Conv. Method (CF)	SBVR Method (CF/ SBVR)	Conv. Method (CF)	SBVR Method (CF/ SBVR)	Conv. Method (CF)
26.52 (22.7 cc)	3.40% (4.52/18.40)	− 11.31% (3.92)	4.51% (4.64/22.64)	− 13.02% (3.92)	4.92% (4.64/25.51)	− 12.53% (3.92)
11.49 (10.3 cc)	2.14% (3.82/36.04)	− 17.54% (3.19)	0.78% (3.82/36.56)	− 19.17% (3.19)	1.96% (3.82/36.50)	− 17.44% (3.19)
5.71 (6.36 cc)	− 11.88% (2.70/12.68)	− 14.43% (2.64)	− 8.31% (2.70/13.0)	− 10.77% (2.64)	− 9.24% (2.67/12.66)	− 10.26% (2.64)
Liver (~ 1600 cc)	− 1.35% (4.34/2.21)	11.86% (5.0)	− 0.30% (4.34/2.20)	13.70% (5.0)	3.20% (4.52/2.30)	15.75% (5.0)
Kidney R (~ 168 cc)	3.00% (3.80/5.0)	26.32% (5.0)	3.00% (3.80/5.1)	26.28% (5.0)	− 2.59% (3.84/5.74)	21.20% (5.0)
Kidney L (~ 170 cc)	− 5.06% (3.76/4.72)	20.98% (5.0)	− 8.98% (3.77/4.81)	17.82% (5.0)	− 6.96% (3.79/4.97)	18.92% (5.0)

*VOI*, Volume of interest; *CF*, Calibration factor [﻿cps/MBq]; and *SBVR*, Sphere-to-background counts/voxel ratio

## Discussion

In this study, we showed that the spheres/lesions surrounding background has an important effect on the quantification accuracy. The effect of the surrounding background on the calculation of a CF can be seen and understood from the validation of the NEMA Val. acquisition. When comparing the quantification errors between the conventional method and the SBVR method, these were comparable for the two largest spheres but vary significantly for the small sphere (Table 3). This indicates that background effects for small VOIs are stronger. When not taking into account the background effects, significantly higher quantification errors occur (e.g. quantification errors of 30.28%, 29.19% and 24.32% for different time per view settings for the small sphere in Table 3). The SBVR method could be used routinely in patients by drawing organ and tumor VOIs and their corresponding surrounding background VOIs. The CF map would be then used to obtain the corresponding CF for a given organ or tumor. Calibration factors obtained at different time point acquisitions from the map for a given organ or tumor would allow to calculate the time activity curve for the corresponding organ/tumor and to calculate radiation absorbed dose. For bone marrow, the absorbed dose is calculated from blood samples drawn at different time points and not from imaging.

The maximum CF value for the SBVR method was obtained for a VOI volume large enough, so the PVE was negligible, and for no background effects (cold background). Those conditions were met by the PET cylinder acquisition (conventional method) where the VOI was delineated across the entire phantom cavity (5600 cc) and the surrounding background was cold. The obtained background CF of 5.0 ﻿cps/MBq was then used for quantification of large organs without accounting for the background effects in scope of the conventional method. In the SBVR method, the background CF (maximum CF value) was obtained using rectangle VOIs delineated along the length of the NEMA Cal. 2 acquisitions with activity in the background. A background CF of 4.87 was obtained, compared to CF 5.0 obtained with the PET cylinder differing by only 2.6%. Therefore, the use of the NEMA IQ image quality body phantom is feasible not only to calculate CFs for different sphere volumes but also for large volume background, eliminating the need for using a separate phantom (cylindrical phantom) to derive the background CF, as usually proposed in the literature [13].

The background in the NEMA Val. and NEMA Cal. 1 acquisitions was comparable (0.14 vs 0.16 MBq/cc). Despite this, the conventional method failed to provide an acceptable quantification error for the small sphere, with an error margin of 30%, compared to the SBVR method with an error margin lower than of 10% for 30-s per view. The background in the anthropomorphic phantoms is not uniform, and VOIs may be partially bordering areas with different activity concentrations. For example, in Fig. 3b the small sphere is surrounded on one side by the phantom background and on the other side by air (phantom fringes). This had noticeable effects on the quantification using the conventional method and much less with the SBVR method. The background effects on the CF values were explored using the CF map (Fig. 6 and Additional file 1: Table S2), and difference between conventional CF and SBVR CF values up to 40% was obtained.

In addition, the quantification errors in the anthropomorphic phantoms for the conventional method were greater than the ones obtained using the SBVR method, as demonstrated in Tables 4 and 5. The largest errors in the Torso Val. acquisitions were obtained for the small sphere (23.42% compared to 9.34% for the conventional and SBVR methods, respectively) as well as for large organs in the liver/kidney Val. acquisition (26.32% compared to 3% for the right kidney using conventional and SBVR methods, respectively). This shows that the background effects depend on the uniformity of the surrounding background, as well as the VOI volume. Therefore, in addition to lesions SBVR also plays an important role in the quantification of large organs.

The maximum CF (4.87 ﻿cps/MBq) can also be achieved for low SBVR values (non-negligible background effects) with a sufficiently large VOI. However, for a small VOI and a large SBVR it is not possible to obtain the maximum CF due to the presence of the PVE. These conclusions are consistent with results reported by Johannes Tran-Gia et al. [21] in the multi-national evaluation of the accuracy of quantitative ^177^Lu SPECT/CT imaging. In this study, multiple recovery coefficient (RC) curves were obtained for different camera models and none of the maximum RC values converge on 1.0 due to the presence of background effects and different SBVR values.

The inclusion of separation areas between the delineated VOI and the background is essential; it prevents spillover between the VOI and the background and ensures that inaccuracies in the delineation process do not affect the quantification error. For example, in kidneys VOI without a separation area the background VOI would include some of the kidney VOI that was not delineated, increasing the quantification error (Fig. 7a). However, by delineating a separation area, the additional counts will not be added to the background VOI. SBVR was superior to SBAR for modeling the background effects. This can be attributed to the fact that SBAR values are calculated by measuring the activity concentrations directly from the phantom, independent from camera output, whereas SBVR values are calculated using data obtained from the output of the camera (counts per voxel). Moreover, the SBVR method produces feasible quantification errors for shorter time per view settings. This has the potential to reduce imaging times without a significant increase in quantification error. The new quantification method developed here, the SBVR method, is simple to implement and may enable standardization of the gamma camera calibration procedure.

In this study, only the three largest spheres were used in the calibration process. This limitation is induced by the GE Xeleris 3.0 DTK software that does not allow for a robust and continuous drawing of sphere VOIs, even on CT images. For example, the 5.71 cc sphere could be delineated either with a sphere VOI of 6.36 cc or 4.50 cc (see Tables 2, 3, 4, 5), adding uncertainty to the delineation process, which increases with small volumes. CF calculations were therefore performed only for the largest spheres in order to minimize the delineation uncertainty. CF values for volumes smaller than 6.36 cc can be obtained by extrapolating to the CF versus VOI volume curve to small volumes (Fig. 5).

A second limitation of this study was the lack of larger spheres in the calibration phantom (NEMA IQ). The largest sphere used in this study was 26.52 cc, and as such it was impossible to investigate the background effects for spheres of larger volumes by direct measurement. This would allow to confirm the background effects obtained in this study for large VOIs surrounded by uniform activity. The SBVR should also be evaluated for more complex anthropomorphic phantoms such as the SPECT Thorax Phantom, which includes full anatomy with different fillable organs (kidneys, liver and spleen) and tumor inserts, adding in the complexity of background effects. Finally, the SBVR method needs to be evaluated for different camera types, by constructing a CF map for the corresponding camera, to verify its versatility.

Another limitation of this study is that we did not take into account the influence of the geometry on CF values as recently shown by Grings et al. [18]. For future studies, it might be beneficial to investigate and incorporate such corrections for organ geometries in the SBVR method.

## Conclusion

The developed SBVR methodology allows to obtain consistent results with acceptable quantification errors for different phantoms, for uniform and non-uniform background. Moreover, the results obtained for shorter time per view settings showed similar quantification results compared to the full-time acquisition which in turn promotes shorter imaging times with ^177^Lu. Furthermore, the proposed method is simple to set up and can be proposed for standardization of the calibration process.

## Supplementary Information

**Additional file 1**: **Table S1**. CF values [﻿cps/MBq] as function of SBVR and SBAR for the 5.71, 11.49 and 26.52 cc spheres. **Table S2**. Derived Calibration Factor map providing the CF values in ﻿cps/MBq for SBVR values and VOI volumes in cc.

## Data Availability

Phantom imaging raw data are stored in the hospital archiving system at the Hadassah-Hebrew University Medical Center, Jerusalem, Israel.

## Abbreviations

OSEM: Ordered subset expectation maximization
SBVR: Sphere-to-background voxel ratio
SBAR: Sphere-to-background activity ratio
CF: Calibration factor
RC: Recovery coefficient
VOI: Volume of interest
DEW: Double energy window
TEW: Triple energy window
PRRT: Peptide receptor radionuclide therapy
SPECT: Single photon emission computed tomography
CT: Computed tomography
