# Supplementary material for: Towards accurate 177Lu SPECT activity quantification and standardization using lesion-to-background voxel ratio
Source: EJNMMI Phys. 2023 Jan 23;10:5. doi: 10.1186/s40658-023-00526-x (PMC9871126; doi:10.1186/s40658-023-00526-x)
Supplement: Supplementary file 1 — Additional file 1: Table S1. CF values [﻿cps/MBq] as function of SBVR and SBAR for the 5.71, 11.49 and 26.52 cc spheres. Table S2. Derived Calibration Factor map providing the CF values in ﻿cps/MBq for SBVR values and VOI volumes in cc. [file 40658_2023_526_MOESM1_ESM.docx]

**Additional file 1: Tables**

**TABLE S1.** CF values as function of SBVR and SBAR for the 5.71, 11.49 and 26.52 cc spheres

| **SBAR** | **SBVR / CF** | | | | | |
| --- | --- | --- | --- | --- | --- | --- |
|  | **26.52 [cc]** | | **11.49 [cc]** | | **5.71 [cc]** | |
|  | **SBVR** | **CF** | **SBVR** | **CF** | **SBVR** | **CF** |
| Cold BG | 20.01 | 4.64 | 16.87 | 3.86 | 17.29 | 3.08 |
| 17 :1 | 14.63 | 4.21 | 11.20 | 3.39 | 7.25 | 2.22 |
| 14 :1 | 11.19 | 4.04 | 8.24 | 3.09 | 5.08 | 1.96 |
| 10 :1 | 8.33 | 3.87 | 5.99 | 2.91 | 3.75 | 1.82 |
| 6 :1 | 4.68 | 3.49 | 3.46 | 2.67 | 2.43 | 1.78 |

**CF**: Calibration factor

**SBVR**: Sphere to Background counts/Voxel Ratio

**TABLE S2.** Derived Calibration Factor map providing the CF values for SBVR values and VOI volumes

| **SBVR**  **VOI  volume [cc]** | **2** | **4** | **6** | **8** | **10** | **12** | **14** | **16** | **18** | **20** | **20+** |
| --- | --- | --- | --- | --- | --- | --- | --- | --- | --- | --- | --- |
| **6** | 1.62 | 1.80 | 1.97 | 2.15 | 2.32 | 2.50 | 2.67 | 2.85 | 2.97 | 2.98 | 2.98 |
| **7** | 1.89 | 2.08 | 2.25 | 2.43 | 2.61 | 2.79 | 2.97 | 3.15 | 3.25 | 3.24 | 3.24 |
| **8** | 2.13 | 2.31 | 2.49 | 2.67 | 2.85 | 3.03 | 3.21 | 3.39 | 3.48 | 3.46 | 3.46 |
| **9** | 2.33 | 2.51 | 2.69 | 2.87 | 3.05 | 3.23 | 3.41 | 3.59 | 3.66 | 3.65 | 3.65 |
| **10** | 2.50 | 2.68 | 2.86 | 3.03 | 3.21 | 3.39 | 3.56 | 3.74 | 3.82 | 3.82 | 3.82 |
| **11** | 2.64 | 2.82 | 2.99 | 3.17 | 3.34 | 3.52 | 3.69 | 3.87 | 3.95 | 3.95 | 3.95 |
| **12** | 2.76 | 2.94 | 3.11 | 3.28 | 3.46 | 3.63 | 3.80 | 3.97 | 4.05 | 4.07 | 4.07 |
| **14** | 2.96 | 3.13 | 3.29 | 3.46 | 3.62 | 3.79 | 3.95 | 4.12 | 4.21 | 4.26 | 4.26 |
| **16** | 3.10 | 3.26 | 3.42 | 3.58 | 3.74 | 3.90 | 4.05 | 4.21 | 4.32 | 4.40 | 4.40 |
| **18** | 3.20 | 3.36 | 3.51 | 3.67 | 3.82 | 3.97 | 4.12 | 4.27 | 4.40 | 4.50 | 4.50 |
| **20** | 3.28 | 3.43 | 3.58 | 3.73 | 3.87 | 4.02 | 4.17 | 4.32 | 4.45 | 4.57 | 4.57 |
| **22** | 3.33 | 3.48 | 3.62 | 3.77 | 3.91 | 4.06 | 4.20 | 4.34 | 4.49 | 4.62 | 4.62 |
| **24** | 3.37 | 3.52 | 3.66 | 3.80 | 3.94 | 4.08 | 4.22 | 4.36 | 4.51 | 4.66 | 4.66 |
| **26** | 3.40 | 3.54 | 3.68 | 3.82 | 3.96 | 4.10 | 4.24 | 4.38 | 4.53 | 4.69 | 4.69 |
| **28** | 3.42 | 3.56 | 3.70 | 3.84 | 3.97 | 4.11 | 4.25 | 4.39 | 4.54 | 4.72 | 4.72 |
| **30** | 3.44 | 3.58 | 3.71 | 3.85 | 3.98 | 4.12 | 4.26 | 4.40 | 4.56 | 4.73 | 4.73 |
| **32** | 3.45 | 3.59 | 3.72 | 3.86 | 3.99 | 4.13 | 4.27 | 4.40 | 4.56 | 4.75 | 4.75 |
| **50** | 3.49 | 3.62 | 3.76 | 3.89 | 4.03 | 4.17 | 4.31 | 4.45 | 4.61 | 4.79 | 4.79 |
| **70** | 3.50 | 3.64 | 3.78 | 3.92 | 4.06 | 4.20 | 4.34 | 4.49 | 4.64 | 4.82 | 4.82 |
| **90** | 3.51 | 3.65 | 3.80 | 3.94 | 4.09 | 4.23 | 4.38 | 4.53 | 4.68 | 4.85 | 4.85 |
| **110** | 3.52 | 3.67 | 3.82 | 3.97 | 4.12 | 4.27 | 4.42 | 4.57 | 4.72 | 4.87 | 4.87 |
| **200** | 3.55 | 3.73 | 3.90 | 4.08 | 4.25 | 4.42 | 4.59 | 4.76 | 4.87 | 4.87 | 4.87 |
| **500** | 3.70 | 3.97 | 4.23 | 4.49 | 4.74 | 4.87 | 4.87 | 4.87 | 4.87 | 4.87 | 4.87 |
| **800** | 3.85 | 4.22 | 4.58 | 4.87 | 4.87 | 4.87 | 4.87 | 4.87 | 4.87 | 4.87 | 4.87 |
| **1100** | 4.00 | 4.48 | 4.87 | 4.87 | 4.87 | 4.87 | 4.87 | 4.87 | 4.87 | 4.87 | 4.87 |
| **1500** | 4.22 | 4.86 | 4.87 | 4.87 | 4.87 | 4.87 | 4.87 | 4.87 | 4.87 | 4.87 | 4.87 |

**VOI**: Volume of Interest

**SBVR**: Sphere to Background counts/Voxel Ratio

The blue area represents the maximum CF obtained from the NEMA Cal. 2 backgrounds.
